# Tongue ulcer in a patient with COVID-19: a case presentation

**DOI:** 10.1186/s12903-021-01635-8

**Published:** 2021-05-20

**Authors:** Mohammad Bashir Nejabi, Noor Ahmad Shah Noor, Nahid Raufi, Mohammad Yasir Essar, Ehsanullah Ehsan, Jaffer Shah, Asghar Shah, Arash Nemat

**Affiliations:** 1 Faculty of Dentistry, Kabul University of Medical Sciences, Kabul, Afghanistan; 2 Department of Dermatology, Kabul University of Medical Sciences, Kabul, Afghanistan; 3 Department of Dermatology, Guangdong Provincial Dermatology Hospital, Southern Medical University, Guangzhou, China; 4 Medical Research Center, Kateb University, Kabul, Afghanistan; 5 Department of Dermatology, Alberoni University, Kapisa, Afghanistan; 6 Drexel University College of Medicine, Philadelphia, PA USA; 7 Division of Biology and Medicine, Brown University, Providence, RI USA; 8 Department of Cardiology, Nanfang Hospital, Southern Medical University, Guangzhou, China; 9 Department of Microbiology, Kabul University of Medical Sciences, University Road, Ali Abad, Jamal Mina, 3rd District, Kabul, 1001 Afghanistan

**Keywords:** COVID-19, SARS-CoV-2, Coronavirus, Oral manifestation, Tongue ulcer, Case report

## Abstract

**Background:**

The emergence of COVID-19 has devastated many parts of the world. From asymptomatic to symptomatic, the virus causes a wide spectrum of presentations. COVID-19 patients may present with oral manifestations. In Afghanistan, where COVID-19 has severely strained the health care system, much of the population lacks proper oral hygiene. This makes the oral cavity a perfect site for SARS-CoV-2 to manifest clinical signs.

**Case presentation:**

A 62-year-old male was evaluated in the Dentistry Teaching Clinic of Kabul University of Medical Sciences for a painful erosive lesion on dorsal surface of his tongue. He also complained of fever, cough, and taste alteration. He was referred to Afghan Japan Hospital for COVID-19 testing and tested positive. He was followed on for the treatment of SARS-CoV2. After 2 weeks, the patient tested negative and returned to the dentistry clinic for follow-up. Although there were no other signs of COVID-19, the painful erosive lesion on his tongue persisted. Oral evaluation were performed and the patient was advised to practice good hygiene. After 10 days, we observed an asymptomatic geographic tongue without fever and myalgias and the lesion of dorsal surface of tongue improved from severe condition to moderate.

**Conclusion:**

In conclusion, patients with suspected or confirmed SARS-CoV-2 should be screened for symptoms and physical findings in the oral mucosa To prevent such an outcome, awareness programs need to be implemented for the diagnosis and management of clinical symptoms among patients.

## Background

The novel coronavirus disease 2019 (COVID-19), a viral disease declared a pandemic by the World Health Organization (WHO) in March 2020, has caused a global health crisis affecting tens of millions of people with devastating health and economic consequences [1]. The disease is caused by the novel severe acute respiratory syndrome (SARS) coronavirus-2 (SARS-CoV-2), which causes viral pneumonia [2]. This virus creates widely varied clinical symptoms, from asymptomatic to mild, severe, and critical [3]. Most cases present with mild symptoms, including dry cough, fever, sore throat, nasal congestion, and myalgias. Severe COVID-19 is characterized by severe pneumonia, and critical cases include respiratory failure, septic shock, and multiple organ failure. It has been reported that atypical manifestations could be in some cases the first or only manifestations of this disease [4]. Oral manifestations have been reported in multiple publications [5–7]. The palate and tongue were the most frequent locations, followed by gingiva and lips. Pain was reported by 75% of patients and 25% reported taste alterations [8–13]. Furthermore, the most frequently reported oral manifestations include ulcerative lesions, vesiculobullous/macular lesions, and acute sialadenitis of the parotid gland (parotitis)[14]. The etiology of oral lesions in patients with COVID‐19 is still uncertain and seems to be multi‐factorial. The appearance of such lesions may be related to the direct or indirect action of SARS‐CoV‐2 on the oral mucosa cells, hypersensitivity of drugs used in the treatment of COVID‐19,downgrading of the general state of health of the patient due to the disease and long period of hospitalization [15]. There may be a link between COVID-19 and oral manifestations, but these signs may often go undetected due to a lack of intraoral examination during hospital admission [16]. Therefore, in this article, we aimed to report a case of oral manifestation in a COVID-19 diagnosed patient. This case builds on the findings of previous studies while highlighting the importance of full mouth examination to better understand the pathobiology of these oral alterations.

## Case presentation

On 16th August 2020, a 62-year-old male presented to the Dentistry Teaching Clinic of Kabul University of Medical Sciences, due to a painful erosive ulcer on the dorsal surface of the tongue for one week. The patient reported that two weeks prior he had developed fever, cough, taste alterations, olfactory dysfunction, and chest tightness. He was referred to the local COVID-19 Hospital (Afghan-Japan Hospital) for the treatment of SARS-CoV-2. His rRT-PCR test was positive. He was treated with azithromycin 500 mg daily for one week and ceftriaxone 1 g twice a day for 3 days. After 2 weeks, a repeat COVID-19 test returned negative. When he came to the dentistry clinic, all of his symptoms had resolved except fora painful erosive lesion on the dorsal surface of his tongue. The patient had a history of controlled diabetes mellitus-type-2 and moderate hypertension. He did not have a history of any oral diseases such as candidiasis, lichen planus, or HSV.

Physical examination revealed normal temperature (37C); blood pressure 135/88 mmHg, heart rate 80; respiratory rate 19, and oxygen saturation 98%. We observed a white geographic ulcer with irregular borders on the dorsum of the tongue.

Laboratory examinations showed normal differential leukocyte count (DLC) and total leukocyte count (TLC), C-reactive protein 22.4/L, and glucose 120 mg/L. Computed tomography of lungs showed mild glass ground opacification bilaterally. Polymerase chain reaction (PCR) of a pharyngeal sample detected HSV-1 and he was treated with intravenous Acyclovir 5 mg/kg three times a day for 7 days with no effect on the oral lesions during the treatment.

For pain control of the ulcer, our dentist administered photobiomodulation therapy (PBMT) for 10 days. After 2 days of PBMT, the patient reported relief of symptoms and on the 11th day after PBMT treatment, the lesion partially resolved [17]. Besides the mentioned therapy, we recommended antibiotics (Azithromycin 500 mg for 1 week), antiseptic agents (Chlorhexidine 0.12%, alcohol-free mouth rinses, and H2O2 1%), and antifungal agents (fluconazole 200 mg tablets for 1 week) for the prevention of secondary infections. The patient was advised to avoid hot and spicy foods, but to drink plenty of fluids and eat a bland diet. We also advised the patient to practice good oral hygiene.

**Fig. 1:**
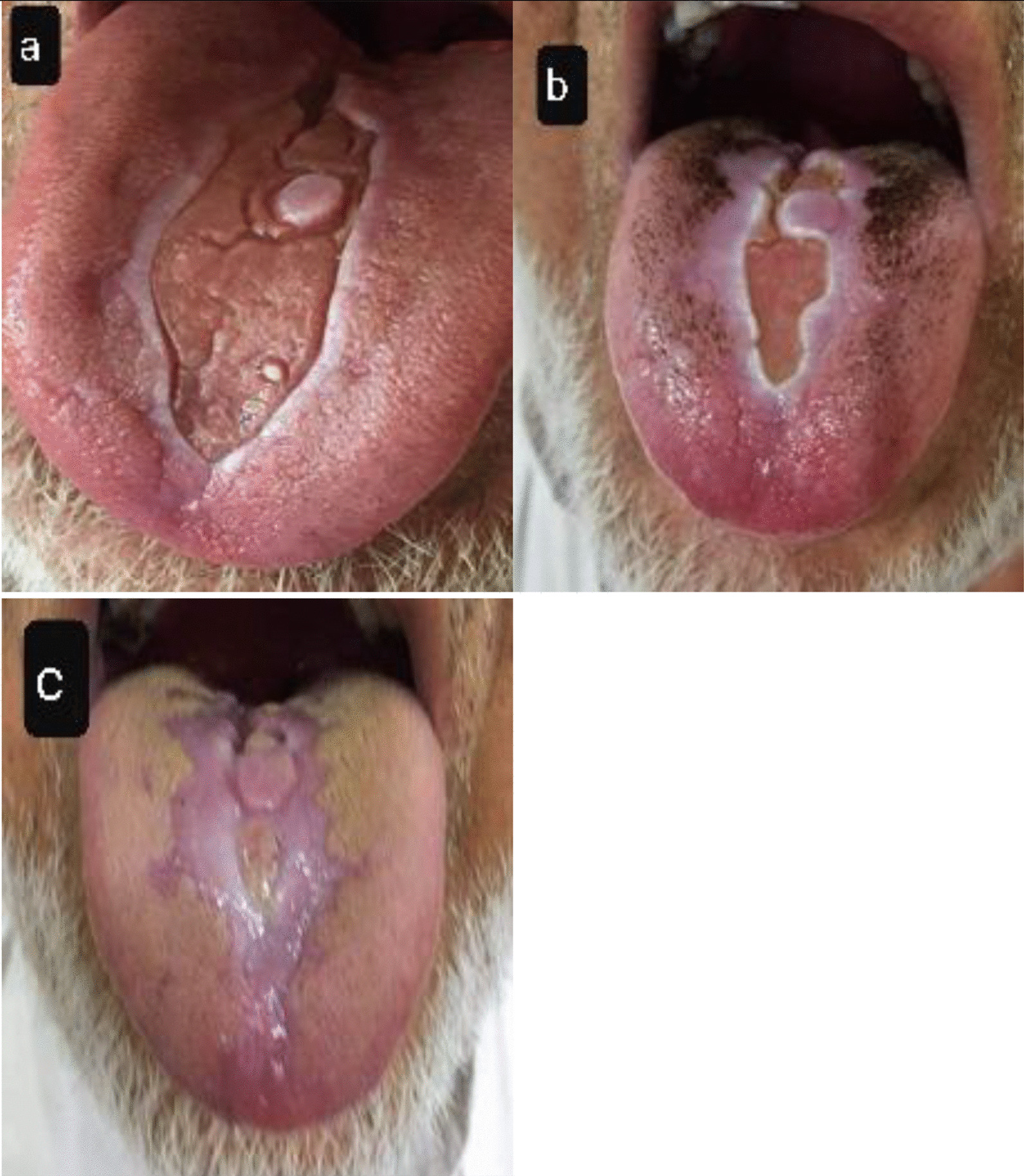
**a** Fissured tongue with white scars after the COVID 19 recovery; **b** After 4 weeks the patient felt better and only he was complaining of burning on his tongue during the eating hot foods; **c** 4 weeks after the first examination

On August 30, 2020, he returned for a follow-up. During the examination, the patient had fewer clinical symptoms, and the size and mass of the lesion were changed to moderate compare to the severe form on the first examination. (Fig. 1).

## Discussion and conclusion

Current research shows that the damage of coronavirus to respiratory and other organs could be related to the distribution of angiotensin-converting enzyme 2 (ACE2) receptors in the human system [18]. Therefore, cells with ACE2 receptor distribution may become host cells for the virus and further cause inflammatory reactions in related organs and tissues, such as the tongue, mucosa and salivary glands. In an analysis of 49 confirmed COVID patients, Zhong and colleagues found high expression of ACE2, and a high detection rate of SARS-CoV-2 RNA in saliva [19]. Moreover, existing evidence has not established an efficient and safe pharmacological agent against COVID-19 yet, and the potential ones are related to several adverse reactions, including oral lesions. Also, COVID-19 acute infection, along with associated therapeutic measures, could potentially contribute to adverse outcomes concerning oral health, likely leading to various opportunistic fungal infections, recurrent oral herpes simplex virus (HSV-1) infection, fixed drug eruptions, dysgeusia, xerostomia linked to decreased salivary flow, ulcerations and gingivitis as a result of the weak immune system and/or susceptible oral mucosa [20, 21]. Moreover, lack of oral hygiene, stress, immunosuppression, vasculitis and hyper-inflammatory response secondary to COVID-19 are some of the major predisposing factors for oral lesions in COVID-19 positive patients [22]. Similar oral conditions were presented by our patient and others have been previously reported [9, 23].

The existing literature on oral manifestations of COVID-19 provides support for our findings, treatment administered, and the ulcer outcome. A review of more than 170 COVID-19 positive cases found changes in tongue sensation and onset of tongue ulceration to be the most common symptoms [24]. The use of photobiomodulation therapy (PBMT) in managing oral lesions has been well documented [25]. Also, the specific use of PBMT as an effective treatment in COVID-19 patients was reported by Soheilifar and colleagues [26]. Prior reporting has indicated improvements in lesion outcomes after treatment. Carreras‐Presas reported three cases of intraoral lesions that were all treated between 3 and 10 days [27].

The occurrence of oral signs and symptoms should be considered in COVID-19 patients, including dysgeusia, petechiae, candidiasis, traumatic ulcers, HSV-1 infection, geographical tongue, thrush-like ulcers, among others. **S**antos and colleagues reported a case of oral mucosal lesions in a COVID-19 patient [28]. Other oral manifestations of the case included recurrent herpes simplex, candidiasis, and benign migratory glossitis. The researchers posit that some oral conditions may be a result of COVID-19 treatment and for this reason, oral health professionals should be included in the clinical care team. A review of 210 COVID-19 cases which reported prone positioning and mechanical ventilation devices in the ICU as resulting in oral mucocutaneous complications reached similar conclusions [29]. Hence, the importance of the clinical examination of the oral mucosa in patients with infectious diseases in the ICU should be emphasized, considering the need for support, pain control, and quality of life. Corchuelo and colleagues report the successful use of teleconsultation as facilitating the interdisciplinary approach for a patient asymptomatic COVID-19 presenting with Candida albicans, thrush, petechiae, and melanin hyperpigmentation at the gingival level [30].

Thorough oral examination, while practicing protective measures to avoid viral transmission, is important in addressing oral manifestations of COVID-19. To that end, Bordea and colleagues report a systematic review of guidelines to provide safe and efficacious oral care during the COVID-19 pandemic [31]. A retrospective study of 47 multisystem inflammatory syndromes in children (MIS-C) positive pediatric patients, who tested positive for COVID-19 infection, concluded that dental care providers play an important role in the early detection of MIS-C and in the identification of oral lesions in MIS-C patients [32]. They posit, furthermore, that MIS-C incidence is likely to increase as the number of COVID-19 positive cases continues to grow. All things considered, oral healthcare providers can play an important role in the detection and subsequent treatment of oral manifestations following COVID-19 infection.

In conclusion, we affirm that the problems that arise in the oral mucosa in patients with suspected or confirmed SARS-CoV-2 infection should be monitored during the pandemic, as demonstrated in our case of a dorsal tongue ulcer in a COVID-19-positive patient. To prevent such an outcome, awareness programs need to be implemented for the diagnosis and management of clinical symptoms among patients.

## Data Availability

The readers can acquire available data and materials in the current study by sending an email to dr.arashnemat@yahoo.com.

## Abbreviation

PCR: Polymerase chain reaction
